# Deceleration Capacity Improves Prognostic Accuracy of Relative Increase and Final Coronary Physiology in Patients With Non-ST-Elevation Acute Coronary Syndrome

**DOI:** 10.3389/fcvm.2022.848499

**Published:** 2022-03-22

**Authors:** Jun Wang, Chengzhe Liu, Fuding Guo, Zhen Zhou, Liping Zhou, Yueyi Wang, Huaqiang Chen, Huixin Zhou, Zhihao Liu, Shoupeng Duan, Ji Sun, Qiang Deng, Saiting Xu, Hong Jiang, Lilei Yu

**Affiliations:** ^1^Department of Cardiology, Renmin Hospital of Wuhan University, Wuhan, China; ^2^Cardiac Autonomic Nervous System Research Centre of Wuhan University, Wuhan, China; ^3^Cardiovascular Research Institute, Wuhan University, Wuhan, China; ^4^Hubei Key Laboratory of Cardiology, Wuhan, China

**Keywords:** deceleration capacity, non-ST-elevation acute coronary syndrome (NSTE-ACS), coronary physiology, major adverse cardiac and cerebrovascular events (MACCEs), quantitative flow ratio (QFR)

## Abstract

**Background:**

Both coronary physiology and deceleration capacity (DC) showed prognostic efficacy for patients with acute coronary syndrome (ACS). This retrospective cohort study was performed to evaluate the prognostic implication of DC combined with the relative increase and final coronary physiology as detected by quantitative flow ratio (QFR) for patients with non-ST-elevation ACS (NSTE-ACS) who underwent complete and successful percutaneous coronary intervention (PCI).

**Methods:**

Patients with NSTE-ACS who underwent PCI with pre- and post-procedural QFR in our department between January 2018 and November 2019 were included. The 24-hour deceleration capacity (DC 24h) was obtained *via* Holter monitoring. The incidence of major adverse cardiac and cerebrovascular events (MACCEs) during follow up was defined as the primary outcome. The optimal cutoffs of the relative increase, final QFR, and DC 24h for prediction of MACCEs were determined *via* receiver operating characteristic (ROC) analysis and the predictive efficacies were evaluated with multivariate Cox regression analysis.

**Results:**

Overall, 240 patients were included. During a mean follow up of 21.3 months, 31 patients had MACCEs. Results of multivariate Cox regression analyses showed that a higher post-PCI QFR [adjusted hazard ratio (HR): 0.318; 95% confidence interval (CI): 0.129–0.780], a higher relative QFR increase (HR: 0.161; 95% CI: 0.066–0.391], and a higher DC (HR: 0.306; 95% CI: 0.134–0.701) were all independent predictors of lower risk of MACCEs. Subsequently, incorporating low DC (≤2.42) into the risk predicting model with clinical variables, the predictive efficacies of low relative QRS increase (≤23%) and low post-PCI QFR (≤0.88) for MACCEs were both significantly improved.

**Conclusions:**

The DC combined with relative increase and final coronary physiology may improve the predictive efficacy of existing models based on clinical variables for MACCEs in NSTE-ACS patients who underwent complete and successful PCI.

## Introduction

Currently, physiological assessment of coronary artery stenosis has become an important standard for decision making in percutaneous coronary intervention (PCI) (1–3). Indeed, it has been shown that up to 20% of patients with successful revascularization as evidenced by angiographic findings still suffer from subsequent adverse coronary events (4–6). Recently studies showed that these patients were likely to have residual or diffuse disease and/or stented segment, due to condition of anatomical revascularization, but not functional revascularization (4–6). Previous studies have shown that the relative increase and final fractional flow reserve (FFR) were reliable parameters for evaluating coronary functional revascularization and may confer prognostic efficacy for patients after PCI (4). Furthermore, recent studies suggest that quantitative flow ratio (QFR), a highly consistent parameter, with FFR indicating functional stenosis of coronary arteries, may also be a validated prognostic index after PCI (5–7). However, the current understanding of coronary artery disease (CAD) indicates that the progression of disease is not only determined by the anatomy or physiology of the coronary lesion alone but is also influenced by systemic factors, such as inflammation and autonomic dysfunction (8–10). In this regard, an integrated approach incorporating parameters of functional revascularization, such as QFR, may confer better prognostic implications in patients with non-ST-elevation acute coronary syndrome (NSTE-ACS) after PCI.

Our previous study revealed a significant association between parameters of heart rate variability (HRV), inflammation, and coronary artery physiology based on QFR (11). Specifically, the 24-hour deceleration capacity (DC 24h), a Holter-derived indicator of parasympathetic activity, has also been suggested as a strong predictor of mortality for patients with myocardial infarction (12). Physiologically, automatic nervous system (ANS) carries the essential function in the formation of the heart and critical regulator of vascular development during cardiovascular development (13). Moreover, pathologically, autonomic function, particularly the activity of the vagus nerve, has been correlated with systemic inflammation (13, 14) and vascular tension (15). Therefore, we hypothesized that an integrated approach incorporating the relative increase and final coronary physiology with DC 24h may improve the prognostic efficacy of current models based on clinical variables in NSTE-ACS patients who underwent complete and successful PCI.

## Methods

### Patient Population

**Figure 1:**
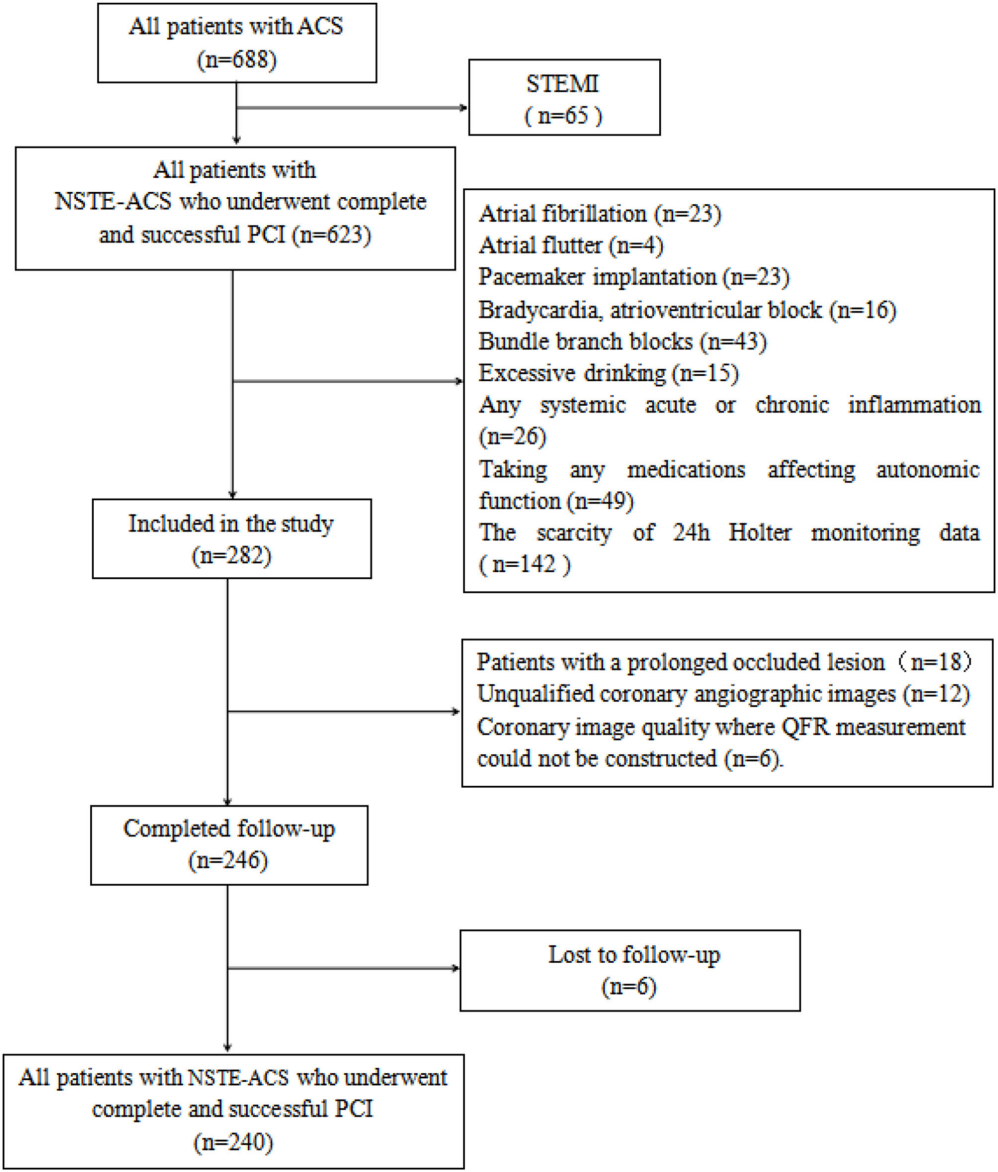
Flowchart of patient inclusion.

Patients with NSTE-ACS who underwent complete and successful PCI with adequate information of pre- and post-PCI QFR computation in the Department of Cardiology of Renmin Hospital of Wuhan University between January 2018 and November 2019 were retrospectively included. The diagnosis of NSTE-ACS was in accordance with the criteria of current guidelines (16), which include unstable angina pectoris (UA) and non-ST segment elevation myocardial infarction (NSTEMI). Complete and successful PCI was defined as the achievement of residual stenosis <20% and final thrombolysis in myocardial infarction (TIMI) flow grade 3. Patients with the following clinical conditions were excluded from the study: atrioventricular block, bundle branch blocks, pacemaker implantation, atrial fibrillation, atrial flutter, chronic coronary syndrome, acute ST-segment elevation myocardial infarction, hyperthyroidism, excessive alcohol intake, any malignancies, any systemic acute or chronic inflammation, use of any medications affecting autonomic function, scarcity of 24h Holter monitoring data, and nonstandard dual-antiplatelet therapy. Patients with the following coronary lesion characteristics which prevented QRS analysis were also excluded: prolonged occluded lesion, coronary bypass graft, left main coronary artery disease, coronary slow flow, unqualified coronary angiographic images including ostial lesion, myocardial bridge, severe vessel overlap or tortuosity at the stenotic segments, and poor coronary image quality where measurement of QFR was not applicable. The study was approved by the Ethics Committee of Renmin Hospital of Wuhan University (No. WDRY2021-K078) before the performance. The flowchart of patient enrollment is shown in Figure 1.

### Holter Monitoring

Before the PCI procedure, all patients received Holter monitor examination for HRV analysis and derivation of DC 24h. Holter monitor data was analyzed as previously described (12, 17–20) to obtain the standard time-domain and frequency-domain parameters. Briefly, R peak detection was used to identify normal sinus RR intervals and then the standard deviation of all normal sinus RR intervals (SDNN), root mean square successive difference (RMSSD), and standard deviation average of NN intervals (SDANN) were calculated. PNN50 represents the percentage of the number of times that the difference between adjacent normal RR intervals is >50 ms over the total number of NN intervals. The high-frequency power (HF) was defined as high frequency spectra 0.15–0.4 Hz; low-frequency power (LF) as frequency spectra 0.04–0.15 Hz; and very low-frequency (VLF) as frequency spectra 0.003–0.04 Hz. Low-frequency/high-frequency (LF/HF) denotes the ratio of the parameters. Normalized LF and HF powers were calculated with the following equations: LFn = 100^*^LF/ (total power-VLF) and HFn = 100^*^HF/ (total power-VLF). DC 24h was calculated, following transformation of the RR intervals using phase-rectified signal averaging, by introducing anchor points (RR0) into the tachygram and generating a plot of all RR intervals recorded. Four-beat segments were defined as two beats prior to and two beats after the anchor points. The preceding RR-intervals, defined as RR-1 and RR-2, and the RR-interval following RR0 (RR + 1) were used in the analysis. The mean values of RR-2, RR-1, RR0 and RR + 1 were used in the equation DC 24h = [X (0) + X (1) – X (−1) – X (−2)] /4 to calculate DC 24h (12, 19, 20).

### QFR Computation

For pre-PCI QFR and post-PCI QFR, analysis of all participants was performed offline and analyzed with the AngioPlus system (Pulse Medical Imaging Technology, Shanghai, China). Two selected views of the same coronary artery greater than 25°were transferred to the QFR system and the QFR was calculated by establishing the contrast flow model. Using a modified TIMI frame count method, the contrast flow rate was estimated from coronary angiography images and the contrast flow model was calculated.

**Figure 2:**
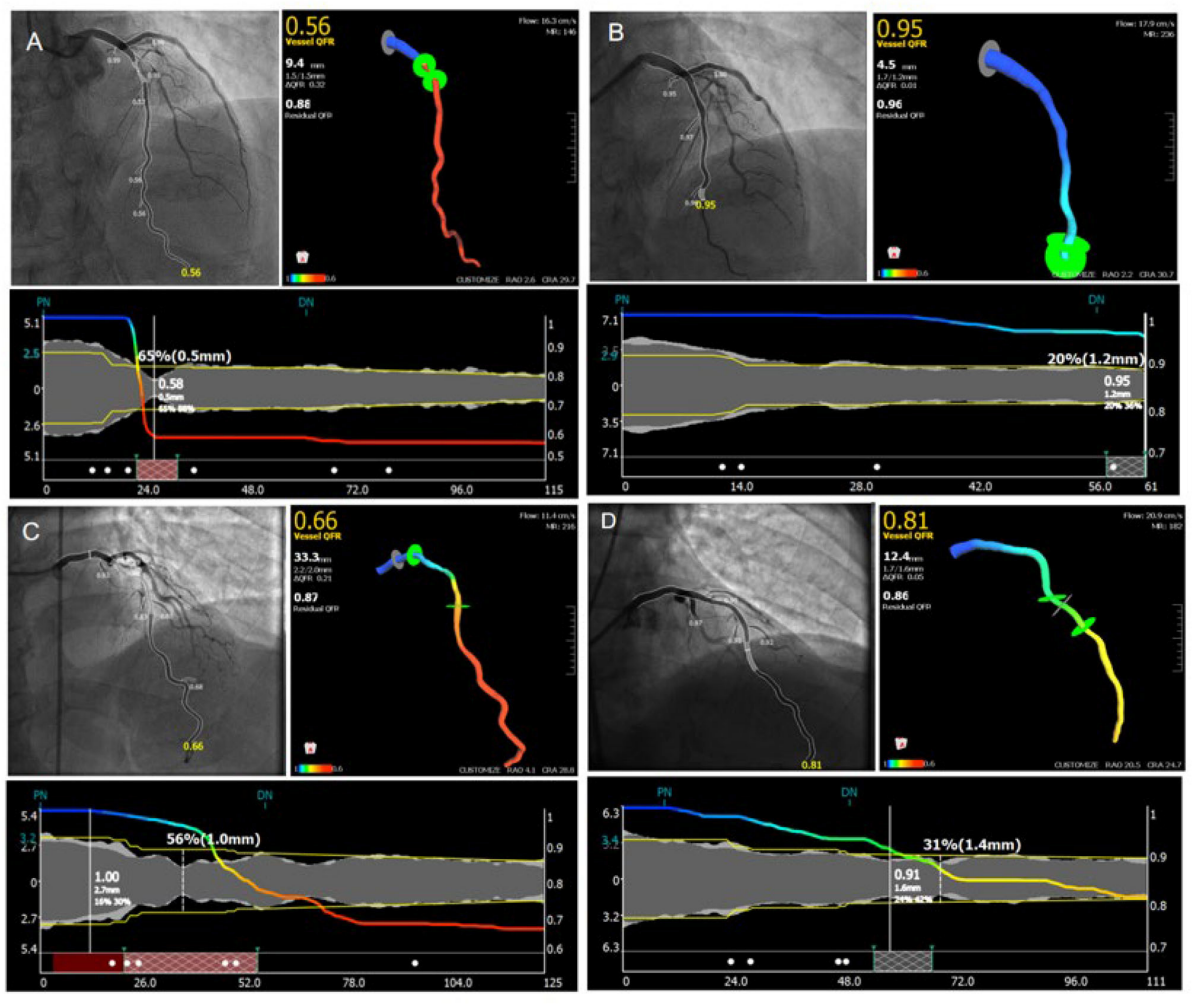
Representative images for cQFR analysis. **(A,C)** Representative images for the measuring of pre-percutaneous coronary intervention quantitative flow ratio. **(B,D)** Representative images for the measuring of post-percutaneous coronary intervention quantitative flow ratio.

In our study, the contrast-flow quantitative flow ratio (cQFR) was derived from routine coronary angiography of the target vessel that was most clinically relevant or with the most severe stenosis. The relative increase of QFR was calculated by %QFR increase with PCI [(post-PCI QFR–pre-PCI QFR) / pre-PCI QFR^*^ 100]. Representative examples are shown in Figure 2.

### Follow Up

Outcome data were obtained either by phone or by clinical visit after discharge. The incidence of major adverse cardiac and cerebrovascular events (MACCEs) during follow up was selected as the primary outcome, which was defined as a composite of cardiac mortality, stroke, revascularization and re-admission for UA. Cardiac mortality was defined as overall mortality from cardiac causes. Stroke was defined as fatal or non-fatal ischemic stroke. Revascularization was defined as revascularization on target or non-target vessels. Re-admission for UA was defined as a new admission for UA following discharge from the index hospitalization for successful PCI.

PCI was performed by an experienced senior interventional cardiologist according to standard procedures with a second-generation drug eluting stent (21). Each patient received a loading dose of either aspirin and clopidogrel prior to PCI. All patients were instructed to take aspirin indefinitely plus a P2Y12 inhibitor for at least 1 year after PCI in conformity with current guidelines with respect to recommended duration of drug therapy (21). After PCI, patients continued with optimized medical treatments and were followed up at clinics regularly after discharge.

### Statistical Analysis

Continuous variables were presented as “mean ± SD” for normal distribution, and medians and interquartile ranges (IQRs) for skewed distribution. A chi-square (χ^2^) test was used to analyze the differences among categorical variables, and comparisons of means among multiple groups were performed with ANOVA. A Mann-Whitney U test or Kruskal-Wallis variance analysis was used for analyzing non-normal distribution. The Kaplan-Meier survival curve was used to analyze the potential associations between DC 24h, post-PCI QFR, and % QFR increase at baseline with the incidence of MACCEs in NSTE-ACS patients with stent placement. Univariate analysis was carried out first, followed by multivariate Cox regression analysis incorporating variables with significant findings in univariate analysis. The predictability of MACCEs using DC 24h, post PCI QFR, % QFR increase and LF/HF by ROC curve analysis. Moreover, comparisons were also performed to evaluate whether adding DC 24h, post PCI QFR and % QFR increase to the classic risk factors for cardiovascular disease could improve the predictive ability of the models. SPSS 23 was applied for the statistical analysis, with *p* < 0.05 indicating statistical significance.

## Results

### Patient Characteristics

A total of 240 patients with NSTE-ACS who underwent PCI were retrospectively included. The mean age of the patients was 62.8 years and 75.4% of them were male. Most (88.3%) of the patients had UA. During a mean follow-up of 21.3 months, 31 patients had MACCEs.

### ROC Analyses for MACCEs

**Figure 3:**
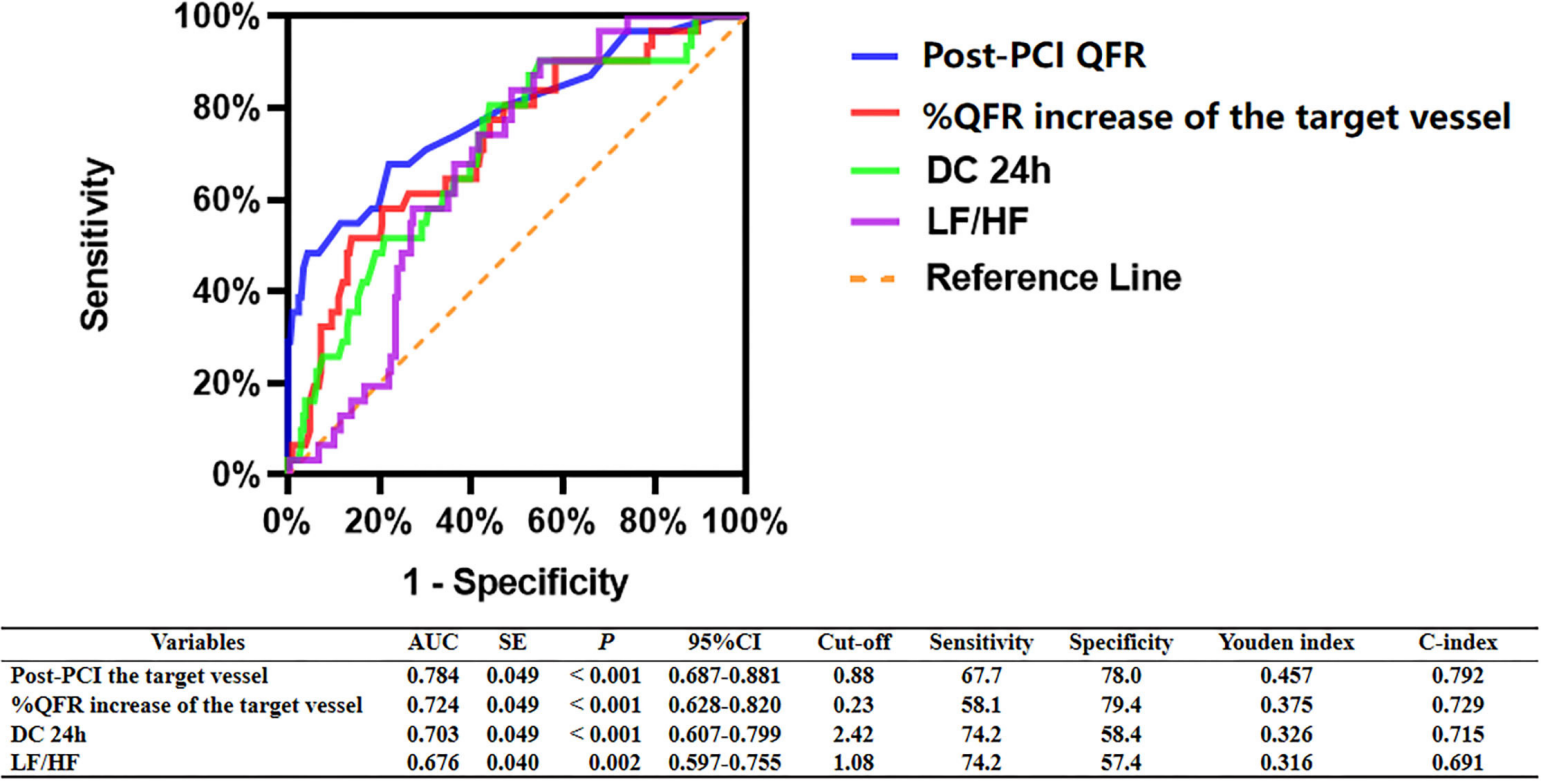
ROC analysis comparing the predictive efficacies of related variables for the incidence of MACCEs during follow up.

As shown in Figure 3, ROC analyses showed that post-PCI QFR, percent QFR increase, DC 24h and LF/HF were all potential predictors for MACCEs, with the optimized cutoff values of 0.88, 23%, 2.42, and 1.08 and the area under the ROC (AUC) of 0.784, 0.724, 0.703, and 0.676, respectively.

### Patient and Target Vessel Characteristics According to DC 24h

As shown in Table 1, patients with lower DC 24h were more likely to be older (*p* < 0.05). Moreover, patients with low DC 24h had higher incidence of MACCEs, revascularization, and re-admission for UA compared to patients with high DC 24h (all *p* < 0.05; Table 2).

**Table 1:** Comparison of patient information and target vessel characteristics according to 24-hour deceleration capacity.

	**DC 24h ≤2.42** **(*n* = 110)**	**DC 24h >2.42** **(*n* = 130)**	**t/Z/χ^2^**	***p***
Male (%)	86 (78.2)	95 (73.1)	0.838	0.360
Age (years)	64.32 ± 10.76	61.58 ± 9.20	2.127	0.034
Hypertension (%)	37 (33.6)	54 (41.5)	1.581	0.209
Duration of Hypertension (years)	11.00 (5.50, 16.50)	10.50 (3.00, 18.00)	0.635	0.525
Diabetes mellitus (%)	33 (30.0)	32 (24.6)	0.875	0.350
Duration of diabetes mellitus (years)	5.00 (3.00, 9.00)	5.00 (2.00, 9.75)	0.751	0.453
Current smoking (%)	55 (50.0)	52 (40.0)	2.412	0.120
Current smoking cigarettes per day	12.50 (6.25, 20.00)	20.00 (10.00, 20.00)	0.408	0.683
Duration of smoking (years)	18.00 (10.00, 23.75)	19.00 (12.25, 26.75)	1.009	0.313
Current drinking (%)	30 (27.3)	26 (20.0)	1.762	0.184
Family history of CAD (%)	11 (10.0)	18 (13.8)	0.830	0.362
History of myocardial infarction	8 (7.3)	11 (8.5)	0.116	0.734
Previous PCI (%)	32 (29.1)	35 (26.9)	0.139	0.709
**Clinical presentation**			0.113	0.737
Unstable angina pectoris	98 (89.1)	114 (87.7)		
NSTEMI	12 (10.9)	16 (12.3)		
**Vessel characteristics**				
Number of diseased vessels	2.13 ± 0.86	2.15 ± 0.82	0.245	0.807
Number of implanted stents	1.75 ± 1.02	1.86 ± 1.04	0.800	0.425
Target vascular location			2.054	0.358
LAD	55 (50.0)	59 (45.4)		
LCX	18 (16.4)	31 (23.8)		
RCA	37 (33.6)	40 (30.8)		
Minimal lumen diameter	0.86 ± 0.40	0.84 ± 0.37	0.421	0.674
Maximum area stenosis	83.94 ± 9.86	84.38 ± 9.70	0.346	0.730
Lesion length of target vessels	17.00 (11.45, 27.70)	15.90 (9.50, 22.70)	1.417	0.156
pre-PCI QFR	0.66 ± 0.09	0.68 ± 0.09	1.742	0.083
% QFR increase	0.33 (0.22, 0.50)	0.33 (0.23, 0.46)	0.244	0.808
Post-PCI QFR	0.92 (0.85, 0.96)	0.94 (0.90, 0.97)	1.934	0.053

*CAD, coronary artery disease; NSTEMI: acute non-ST-segment elevation myocardial infarction; LAD, left anterior descending artery; LCX, left circumflex artery; RCA, right coronary artery; SDNN, standard deviation of all normal sinus RR intervals; RMSSD, root mean square successive difference; SDANN, standard deviation average of NN intervals; PNN50, percentage of the number of times that the difference between adjacent normal RR intervals >50 ms over the total number of NN intervals; HF, high-frequency power; LF, low-frequency power; DC 24h, twenty-four-hour deceleration capacity; PCI, percutaneous coronary intervention; QFR, quantitative flow ratio*.

**Table 2:** Incidence of adverse outcomes according to 24-hour deceleration capacity.

	**DC 24h ≤2.42 (*n* = 110)**	**DC 24h >2.42 (*n* = 130)**	**χ^2^**	***P***
MACCEs, *n* (%)	23 (20.9)	8 (6.2)	11.533	0.001
Cardiac death, *n* (%)	2 (1.8)	1 (0.8)	0.021	0.884
Revascularization, *n* (%)	9 (8.2)	1 (0.8)	6.448	0.011
Stroke, *n* (%)	3 (2.7)	1 (0.8)	0.455	0.500
Re-admission for unstable angina, *n* (%)	10 (9.1)	4 (3.1)	3.923	0.048

### Patient and Target Vessel Characteristics According to Post-PCI QFR

As shown in Table 3, patients with a post-PCI QFR ≤ 0.88 were more likely to have target lesions of the left anterior descending coronary artery, lower LFn, LF/HF, DC 24h, and pre-PCI QFR, and a smaller relative QFR increase compared to patients with post-PCI QFR > 0.88 of the target vessels (all *p* < 0.05). Besides, patients with post-PCI QFR ≤ 0.88 had higher incidence of MACCEs, revascularization and re-admission for UA compared to those with post-PCI QFR > 0.88 of the target vessels (all *p* < 0.05; Table 4).

**Table 3:** Comparison of patient information and characteristics of target vessels in patients with NSTE-ACS according to post-PCI QFR of the target vessel.

	**Low post-PCI ≤0.88 (*n* = 66)**	**High post-PCI >0.88 (*n* = 174)**	**t/Z/χ^2^**	***P***
Male (%)	46 (69.7)	135 (77.6)	1.606	0.205
Age (years)	62.41 ± 10.44	62.99 ± 9.88	0.403	0.687
Hypertension (%)	19 (28.8)	72 (41.4)	3.223	0.073
Duration of hypertension (years)	10.00 (5.00, 18.00)	11.00 (3.25, 16.00)	0.166	0.868
Diabetes mellitus (%)	18 (27.3)	47 (27.0)	0.002	0.968
Duration of diabetes mellitus (years)	5.00 (2.00, 8.00)	5.50 (3.00, 10.00)	1.391	0.164
Current smoking (%)	28 (42.4)	79 (45.4)	0.172	0.679
Current smoking cigarettes per day	10.00 (3.00, 20.00)	20.00 (10.00, 20.00)	0.880	0.379
Duration of smoking (years)	13.50 (9.25, 24.75)	19.00 (14.25, 25.00)	0.979	0.328
Current drinking (%)	12 (18.2)	44 (25.3)	1.350	0.245
Family history of CAD (%)	9 (13.6)	20 (11.5)	0.207	0.649
History of myocardial infarction	4 (6.1)	15 (8.6)	0.430	0.512
Previous PCI (%)	16 (24.2)	51 (29.3)	0.611	0.435
**Clinical presentation**			1.073	0.300
Unstable angina pectoris	56 (84.8)	156 (89.7)		
NSTEMI	10 (15.2)	18 (10.3)		
Average heart rate (beats/min)	71.47 ± 7.12	69.91 ± 6.71	1.577	0.116
SDNN	111.00 (80.00, 127.50)	112.00 (91.00, 135.25)	1.187	0.235
SDANN	95.00 (73.25, 113.50)	91.00 (73.00, 110.00)	1.181	0.238
rMSSD	38.00 (25.00, 67.75)	42.00 (28.75, 63.25)	0.570	0.569
Pnn50	6.00 (2.00, 15.25)	7.50 (3.00, 15.00)	0.711	0.477
Total power (ms^2^)	1,976.45 (1,327.88, 2,842.18)	2,039.40 (1,361.50, 3,278.55)	0.574	0.566
LF (ms^2^)	117.30 (66.38, 196.56)	151.21 (90.13, 265.64)	2.929	0.003
HF (ms^2^)	116.90 (56.28, 248.03)	130.65 (68.38, 244.63)	0.799	0.425
LF/HF	0.92 (0.70, 1.45)	1.32 (0.76, 1.85)	3.015	0.003
DC 24h ms	2.43 ± 1.10	2.95 ± 1.46	2.959	0.004
**Target vessel characteristics**				
Number of diseased vessels	2.21 ± 0.90	2.11 ± 0.81	0.765	0.446
Number of implanted stents	1.88 ± 1.00	1.79 ± 1.05	0.612	0.541
Target vascular location			24.740	<0.001
LAD	48 (72.7)	66 (37.9)		
LCX	10 (15.2)	39 (22.4)		
RCA	8 (12.1)	69 (39.7)		
Minimal lumen diameter	0.79 ± 0.36	0.87 ± 0.39	1.571	0.118
Maximum area stenosis	84.90 ± 8.76	83.91 ± 10.10	0.694	0.488
Lesion length (mm)	18.60 (11.20, 29.40)	15.95 (10.13, 23.53)	1.394	0.163
Pre-PCI QFR	0.64 ± 0.10	0.68 ± 0.08	3.077	0.002
% QFR increase	0.19 (0.13, 0.36)	0.37 (0.26, 0.52)	6.212	<0.001
Post-PCI QFR	0.82 (0.75, 0.86)	0.95 (0.92, 0.97)	11.880	<0.001

**Table 4:** Incidence of adverse outcomes according to post-PCI QFR of the target vessel.

	**Low post-PCI ≤0.88 (*n* = 66)**	**High post-PCI >0.88 (*n* = 174)**	**χ^2^**	***P***
MACCEs, *n* (%)	21 (31.8)	10 (5.7)	28.914	<0.001
Cardiac death, *n* (%)	1 (1.5)	2 (1.1)	0.000	1.000
Revascularization, *n* (%)	6 (9.1)	4 (2.3)	3.958	0.047
Stroke, *n* (%)	1 (1.5)	3 (1.7)	0.000	1.000
Re-admission for unstable angina, *n* (%)	11 (16.7)	3 (1.7)	16.825	<0.001

### Patient and Target Vessel Characteristics According to Relative QFR Increase

As shown in Table 5, patients with smaller %QFR increase were more likely to have lower LFn, LF/HF, maximum area stenosis of the target vessel, post-PCI QFR and higher pre-PCI QFR (all *p* < 0.05). Moreover, those with smaller %QFR increase had a higher incidence of MACCEs and re-admission for UA compared to patients with larger %QFR increase (all *p* < 0.05; Table 6).

**Table 5:** Comparison of patient information and target vessel characteristics according to percent QFR increase of the target vessel.

	**Low %QFR increase ≤23%** **(*n* = 62)**	**High %QFR increase >23%** **(*n* = 178)**	**t/Z/χ^2^**	***P***
Male (%)	47 (75.8)	134 (75.3)	0.007	0.934
Age (years)	61.53 ± 10.54	63.29 ± 9.83	1.188	0.236
Hypertension (%)	20 (32.3)	71 (39.9)	1.137	0.286
Duration of hypertension (years)	12.00 (7.00, 15.75)	10.00 (3.00, 18.00)	0.062	0.950
Diabetes mellitus (%)	19 (30.6)	46 (25.8)	0.537	0.464
Duration of diabetes mellitus (years)	5.00 (2.00, 8.00)	5.00 (3.00, 9.00)	0.737	0.461
Current smoking (%)	30 (48.4)	77 (43.3)	0.490	0.484
Current smoking cigarettes per day	20.00 (10.00, 20.00)	15.00 (5.00, 20.00)	1.124	0.261
Duration of smoking (years)	15.00 (8.75, 23.50)	19.00 (13.75, 25.50)	1.788	0.074
Current drinking (%)	17 (27.4)	39 (21.9)	0.780	0.377
Family history of CAD (%)	8 (12.9)	21 (11.8)	0.053	0.818
History of myocardial infarction	6 (9.7)	13 (7.3)	0.356	0.551
Previous PCI (%)	16 (25.8)	51 (28.7)	0.185	0.667
**Clinical presentation**			2.994	0.084
Unstable angina pectoris	51 (82.3)	161 (90.4)		
NSTEMI	11 (17.7)	17 (9.6)		
Average heart rate (beats/min)	71.29 ± 7.29	70.01 ± 6.68	1.268	0.206
SDNN	113.50 (81.50, 136.00)	110.00 (90.75, 134.25)	0.120	0.904
SDANN	99.50 (73.25, 120.50)	90.50 (73.00, 109.25)	1.667	0.096
rMSSD	41.00 (25.75, 61.50)	41.50 (28.75, 66.00)	0.681	0.496
Pnn50	7.00 (2.75, 18.25)	7.00 (3.00, 15.00)	0.332	0.740
Total power (ms^2^)	1,933.05 (1,290.48, 2,820.45)	2,039.40 (1,361.50, 3,251.30)	0.801	0.423
LF (ms^2^)	119.65 (53.06, 212.74)	144.25 (93.51, 246.23)	2.658	0.008
HF (ms^2^)	109.60 (60.85,227.50)	131.20 (67.78, 252.13)	0.943	0.346
LF/HF	0.92 (0.62, 1.50)	1.28 (0.76, 1.82)	2.618	0.009
DC 24h ms	2.84 ± 1.50	2.79 ± 1.35	0.214	0.830
**Target vessel characteristics**				
Number of diseased vessels	2.15 ± 0.88	2.14 ± 0.82	0.038	0.970
Number of implanted stents	1.77 ± 0.95	1.83 ± 1.06	0.339	0.735
Target vascular location			3.776	0.151
LAD	35 (56.5)	79 (44.4)		
LCX	13 (21.0)	36 (20.2)		
RCA	14 (22.6)	63 (35.4)		
Minimal lumen diameter	0.87 ± 0.38	0.84 ± 0.38	0.549	0.583
Maximum area stenosis	81.43 ± 9.07	85.11 ± 9.83	2.552	0.011
Lesion length of target vessels	16.30 (11.30, 25.93)	16.40 (10.20, 25.90)	0.625	0.532
Pre-PCI QFR	0.72 ± 0.08	0.65 ± 0.08	5.989	<0.001
% QFR increase	0.16 (0.12, 0.20)	0.40 (0.31, 0.55)	11.473	<0.001
Post-PCI QFR	0.86 (0.80, 0.91)	0.94 (0.91, 0.97)	7.146	<0.001

**Table 6:** Incidence of adverse outcomes according to percent QFR increase of the target vessel.

	**Low %QFR increase ≤23%**	**High %QFR increase >23%**	**χ^2^**	***P***
	**(*n* = 62)**	**(*n* = 178)**		
MACCEs, *n* (%)	18 (29.0)	13 (7.3)	19.301	<0.001
Cardiac death, *n* (%)	1 (1.6)	2 (1.1)	0.000	1.000
Revascularization, *n* (%)	5 (8.1)	5 (2.8)	2.001	0.157
Stroke, *n* (%)	2 (3.2)	2 (1.1)	0.289	0.591
Re-admission for unstable angina, *n* (%)	10 (16.1)	4 (2.2)	13.704	<0.001

### Association Between Relative Increase and Final QFR, and DC for MACCEs

**Figure 4:**
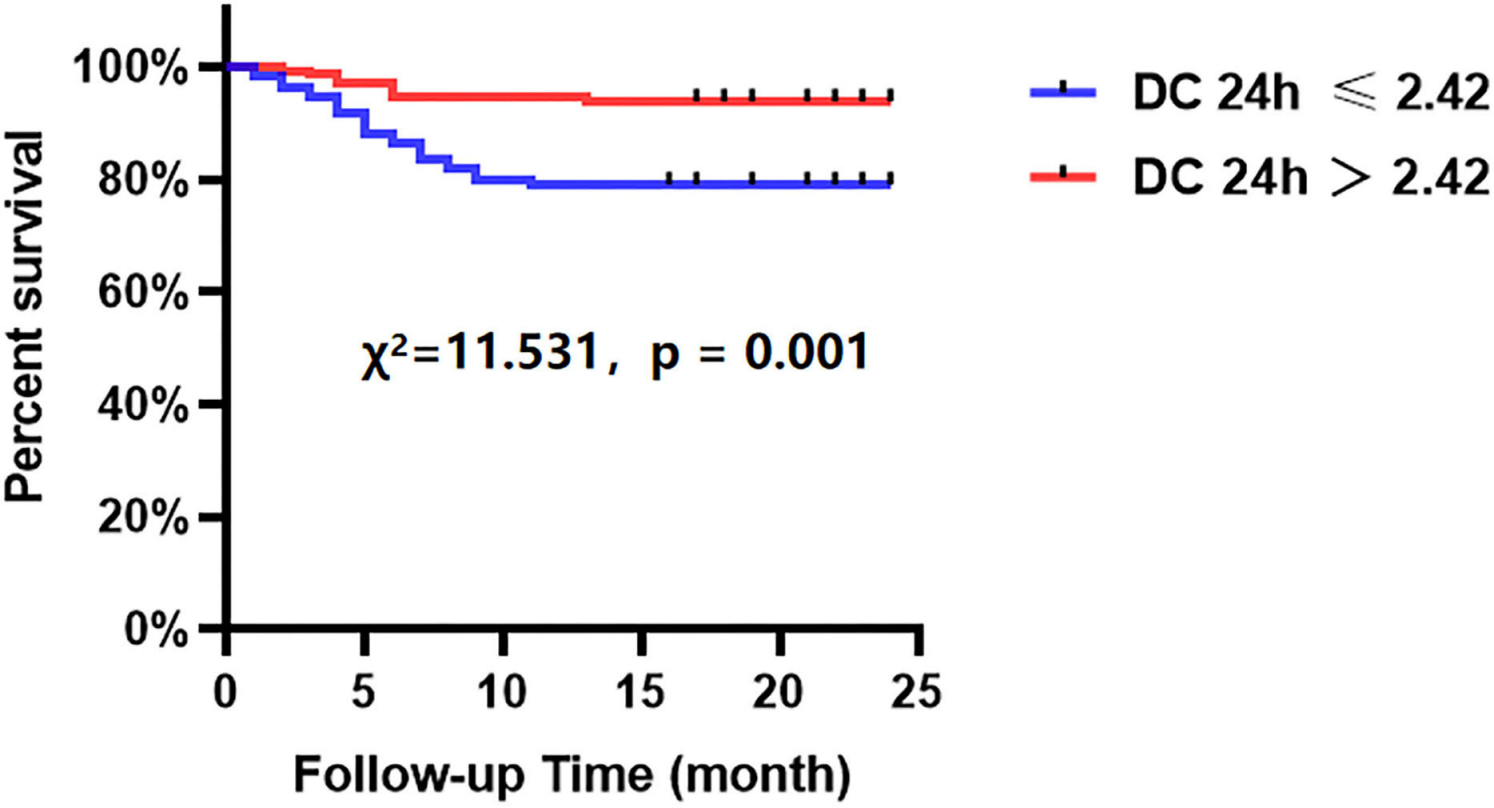
Cumulative event-free survival probability of MACCEs in patients with NSTE-ACS who underwent PCI according to the DC 24 h.

**Figure 5:**
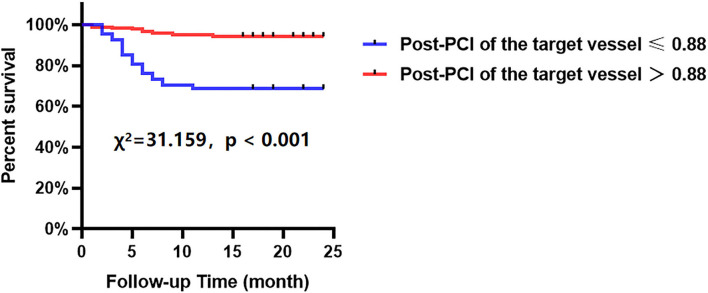
Cumulative event-free survival probability of MACCEs in patients with NSTE-ACS who underwent PCI according to post-PCI of the target vessel.

**Figure 6:**
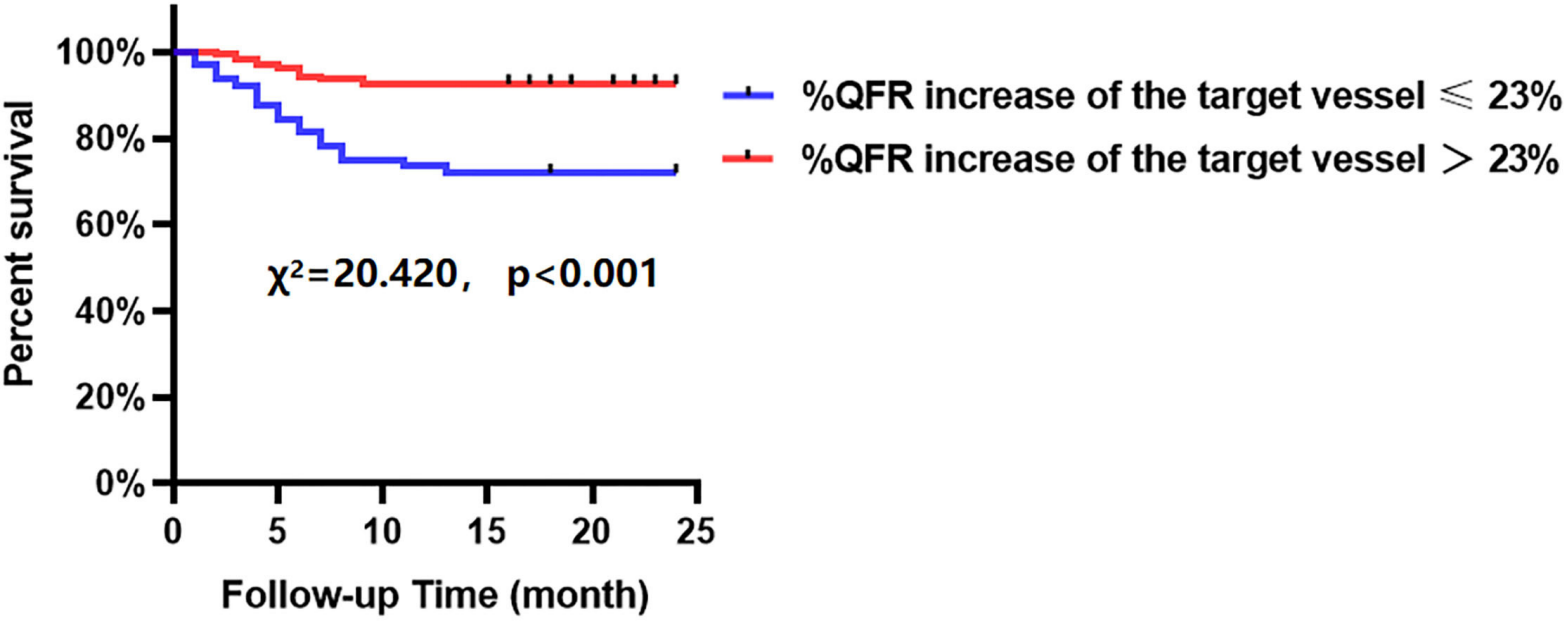
Cumulative event-free survival probability of MACCEs in patients with NSTE-ACS who underwent PCI according to %QFR increase of the target vessel.

The Kaplan-Meier analyses showed that the incidence of MACCEs was significantly different between patients with lower and higher of DC 24h (cutoff: 2.42, χ^2^ = 11.531, *p* = 0.001, Figure 4), post-PCI QFR (cutoff: 0.88, χ^2^ = 31.159, *p* < 0.001, Figure 5), and %QFR increase of the target vessel (cutoff: 23%, χ^2^ = 20.420, *p* < 0.001, Figure 6). Results of multivariate Cox regression analyses suggested that hypertension (HR: 6.816; 95% CI: 2.986–15.559), LF/HF >1.08 (HR: 0.335; 95% CI: 0.144–0.779), DC 24h >2.42 ms (HR: 0.306; 95% CI: 0.134–0.701), pre-PCI QFR (HR: 0.527; 95% CI: 0.346–0.802), post-PCI QFR > 0.88 (HR: 0.318; 95% CI: 0.129–0.780) and relative increase percentage of QFR >23% (HR: 0.161; 95% CI: 0.066–0.391) were all independent predictors of MACCEs (Table 7, all *p* < 0.05).

**Table 7:** Potential predictors for the incidence of MACCEs in patients with NSTE-ACS who underwent PCI.

**Variables**	**Univariate**			**Multivariate**		
	**HR**	**95% CI**	***P***	**HR**	**95% CI**	***P***
Female	1.305	0.601–2.834	0.501			
Age (years)	0.976	0.943–1.010	0.163			
Hypertension (%)	2.425	1.188–4.950	0.015	6.816	2.986–15.559	<0.001
Diabetes mellitus (%)	1.804	0.876–3.717	0.110			
Current smoking (%)	1.022	0.504–2.073	0.952			
Current drinking (%)	0.972	0.419–2.257	0.948			
Family history of CAD (%)	0.783	0.238–2.576	0.688			
History of myocardial infarction	1.678	0.587–4.797	0.334			
Previous PCI (%)	1.427	0.684–2.979	0.343			
Average heart rate (beats/min)	1.035	0.988–1.085	0.147			
SDNN	1.003	0.996–1.011	0.412			
SDANN	1.006	0.995–1.018	0.276			
rMSSD	0.996	0.987–1.005	0.399			
Pnn50	1.012	0.991–1.034	0.246			
Total power (ms^2^)	0.997	0.975–1.019	0.795			
LF (ms^2^)	1.048	0.895–1.226	0.562			
HF (ms^2^)	1.088	0.978–1.210	0.122			
High LF/HF (>1.08)	0.277	0.124–0.620	0.002	0.335	0.144–0.779	0.011
High DC 24h (>2.42 ms)	0.274	0.123–0.613	0.002	0.306	0.134–0.701	0.005
Number of diseased vessels	1.077	0.701–1.653	0.736			
Number of implanted stents	0.989	0.701–1.395	0.949			
Pre-PCI QFR	0.660	0.443–0.984	0.041	0.527	0.346–0.802	0.003
High post-PCI of the target vessel (>0.88)	0.156	0.073–0.331	<0.001	0.318	0.129–0.780	0.012
High %QFR increase of the target vessel (>23%)	0.224	0.110–0.458	<0.001	0.161	0.066–0.391	<0.001
Target vascular location	0.748	0.488–1.145	0.181			
Minimal lumen diameter of target vessel	1.869	0.751–4.647	0.179			
Maximum area stenosis of target vessel	0.982	0.950–1.016	0.295			
Lesion length of target vessels	1.002	0.968–1.036	0.924			

### Prognostic Implication of Relative Increase and Final QFR Combined With DC

**Figure 7:**
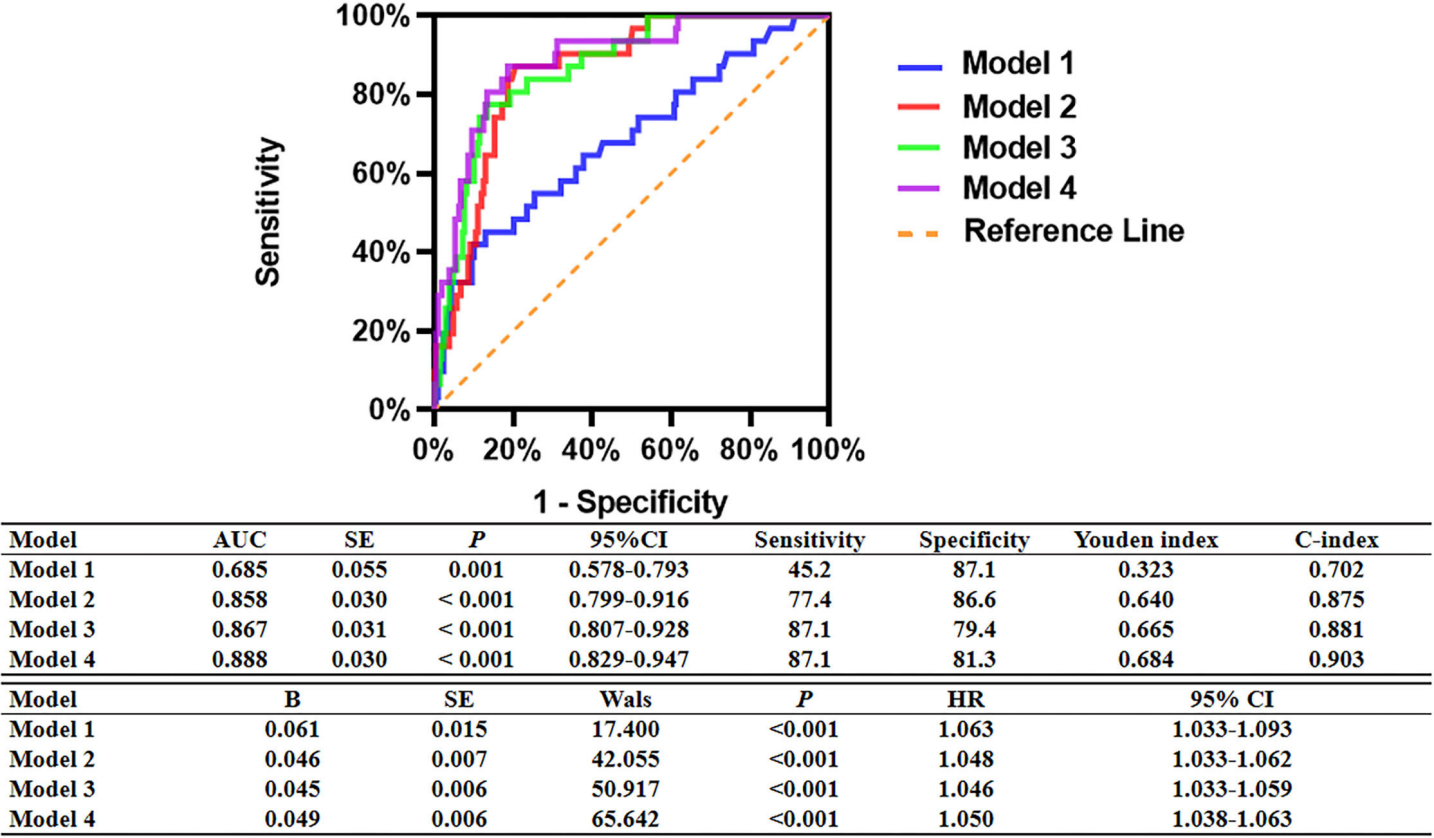
Comparison of the predictive capacity and accuracy of predictive models for MACCEs. Model 1: Age + Sex+ Hypertension + Diabetes mellitus + Current smoking + Family history of CAD + History of myocardial infarction. Model 2: Model 1 + Post-PCI QFR of the target vessel ≤0.88. Model 3: Model 2 + %QFR increase of the target vessel ≤23%. Model 4: Model 3 + DC 24 h ≤2.42 ms.

**Figure 8:**
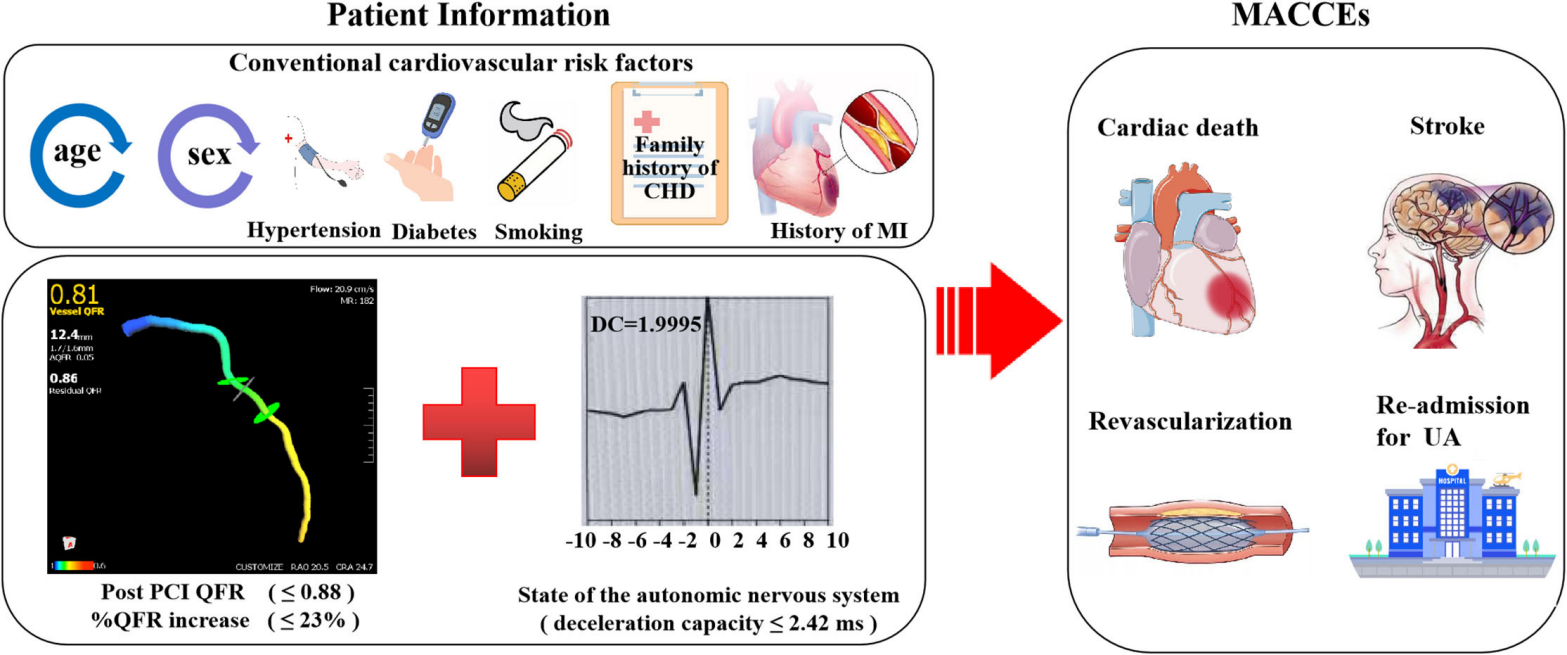
Combined efficacies of post-PCI QFR, %QFR increase and deceleration capacity for risk stratification in patients with NSTE-ACS.

As shown in Figure 7, incorporating post-PCI QFR (Model 2) significantly enhanced the ability to predict accurately the MACCEs compared with Model 1 which included traditional cardiovascular risk factors only (AUC: 0.858 versus 0.685). The predictive ability further increased in Model 3, which incorporated % QFR increase (AUC: 0.867; C-index: 0.881; Youden index: 0.665; sensitivity: 87.1%; specificity: 79.4%; *p* < 0.001). Moreover, Adding DC 24h ≤2.42 into Model 4 further improved the predictive efficacy of the model for MACCEs (AUC: 0.888; C-index: 0.903; Youden index: 0.684; sensitivity: 87.1%; specificity: 81.3%; CI: 0.829–0.947; *p* < 0.001, Figure 8).

As shown in Supplementary Figure 1, the C-index was 0.875 (95% CI: 0.799–0.916, *p* < 0.001) for prognostic model 5, containing model 1 plus post-PCI QFR of the target vessel ≤ 0.88, and 0.802 (95% CI: 0.695–0.877, *p* < 0.001) for model 6, containing model 1 plus %QFR increase of the target vessel ≤ 23%. For model 7, containing model 1 plus DC 24h ≤2.42 ms, the C-index was 0.793 (95% CI: 0.689–0.865, *p* < 0.001). These results suggest that model 5 is a more powerful predictor of MACCEs than %QFR increase of the target vessel ≤23% or DC 24h ≤2.42 ms.

## Discussion

In this retrospective cohort study, we included patients with NSTEMI who underwent complete revascularization with PCI and who had adequate information on pre- and post-procedural QFR and Holter-derived HRV data. We found that lower post-PCI QFR (≤0.88), smaller % QFR increase (≤23%), and lower DC 24h (≤2.42) at baseline were all independent predictors for the risk of MACCEs during follow up. Moreover, incorporation of relative increase and final QFR combined with DC may improve the predictive efficacy of existing models based on clinical variables for MACCEs in patients with NSTE-ACS.

Risk stratification remains challenging in patients with NSTE-ACS, particularly for those after PCI (22, 23). Although multiple large observational studies have suggested a potential role of post-PCI FFR as a predictor for adverse events in the future, the optimal cutoff remains unknown and may be variable according to the different patient populations included (24–26). More importantly, FFR can only be obtained *via* invasive procedures with the assistance of an additional pressure guidewire and the use of adenosine, which significantly limits its use in clinical practice (27). In comparison, QFR, as a noninvasive angiographically-derived FFR measurement is more applicable in clinical settings. Previous studies have confirmed that QFR is highly consistent with FFR and could be used as a validated indicator of functional coronary stenosis (28, 29). A previous study that included 602 patients who underwent complete and successful revascularization with a mean follow-up of 629 days showed that lower values of QFR might be a risk factor for the increased incidence of vessel-oriented adverse events (5). Alternatively, a retrospective study that included 771 vessels with post-PCI QFR suggested a predictive efficacy and independent correlation between post-PCI QFR and long-term vessel-related clinical outcomes in state of the PCI practice (6). Our results, which showed a possible prognostic role of post-PCI QFR for MACCEs in NSTE-ACS patients after PCI, is consistent with the findings of these studies. Nevertheless, neither pre-PCI FFR nor post-PCI QFR alone could fully discriminate the degree of relative contribution of stented and non-stented segment disease burden. Therefore, in order to discriminate the relative contribution of each component of coronary artery lesions, previous studies showed that the percentage of increase of the FFR value before and after PCI has also been independently and significantly correlated with poor long-term prognosis (4), and integration of the concept of percent FFR increase and post-PCI FFR value allowed a better discrimination of high-risk patients after complete and successful PCI (4). In our study, significant associations were observed between percent QFR increase and post-PCI QFR with MACCEs after PCI. However, all these findings are focused on the local interaction between stents and targeted lesions. It could be hypothesized that incorporating parameters that indicated the systematic burden of CAD, such as systematic inflammation and autonomic dysregulation, may further improve the prognostic efficacy of models based on current clinical variables.

Previous studies showed that the ANS is directly involved in cardiovascular development (30–32). Previous ex vivo studies showed that the imbalance of ANS may be an early marker of acute cardiovascular disease events (33–36). In addition, ANS is shown to have key effects on vasoconstriction and vasodilation, which influence vessel physiology (13, 37). Alternatively, the mechanism underlying persistently lowered shear stress appears to be the formation of vulnerable plaque (38). However, there remains scant evidence from literature to indicate the potential association between ANS, hydrodynamic shear forces and the vulnerability of coronary plaque. Our results therefore support the hypothesis that imbalance in cardiac ANS may affect local hydrodynamic shear forces and lead to vulnerability of coronary lesions, and may therefore play a key role in the pathogenesis of acute coronary events. Interestingly, our previous studies showed a significant interaction between ANS and immune inflammation on coronary physiology evaluated by QFR (11, 39). These findings support the incorporation of ANS imbalance for risk stratification of patients with CAD. Notably, measurement of DC is refractory to external factors and premature beat, which could therefore objectively reflect the modulation of heart rate by the ANS and quantitatively analyze vagal nerve activity compared with HRV (12, 19, 20).

Our overall findings are in line with a previous prospective study including 2,111 patients with acute myocardial infarction, which demonstrated that impaired DC was a strong predictor of mortality after myocardial infarction, and the predictive efficacy of DC was even stronger than the conventional measures of HRV (12). In this study, we found that incorporation of relative increase and final QFR combined with DC is associated with stronger predictive efficacy of existing models based on clinical variables for MACCEs in patients with NSTE-ACS. Briefly, acute myocardial ischemia might impair cardiac vagal nerve and induce the production of inflammatory cytokines (14), and subsequently lead to endothelial dysfunction, lipid dysregulation, vascular smooth muscle cell activation, macrophage infiltration, thus accelerating coronary artery plaque rupture of residual lesions and effects on the burden of coronary heart disease (40, 41). Although the exact molecular pathways remain to be determined, the results of our study support the incorporation of relative increase and final QFR combined with DC to improve the predictive efficacy of existing models based on clinical variables for MACCEs in patients with NSTE-ACS.

## Study Limitations

Due to the retrospective nature of the study, possible selection bias may overestimate the predictive value of DC 24h, post-PCI QFR or %QFR increase for MACCEs, particularly with the small sample size. In order to validate the predictive value of these parameters, prospective cohort studies with larger samples sizes will be required. Also, we evaluated the DC only once, on patient admission. It is unclear whether DC monitored for multiple times would be more valid as a predictor of MACCEs. In addition, it should be mentioned that the post-PCI QFR or %QFR increase has not yet been validated against post-PCI FFR or %FFR increase. Studies are warranted for further confirmation and optimization of the software and protocols for QFR measurement. Moreover, our findings might not be generalizable to other institutions due to the differences between offline and online analyses. Nevertheless, the concept regarding that “higher is better” for DC 24hr, post-PCI QFR or %QFR increase when undergoing complete revascularization with PCI is beyond doubt. In addition, intravascular ultrasound or optical coherence tomography may be considered to provide detailed assessment of culprit lesions and non-culprit lesions of the patients, and it might also improve the prognostic efficacy if parameters related to these examinations are incorporated. Due to methodological reasons, DC could not be applied to ACS patients with atrial fibrillation or other non-sinus rhythms. Finally, no inflammatory markers were analyzed because we did not measure these markers in the included patients.

## Conclusions

In conclusion, the results of our study showed that the relative increase and final coronary physiology combined with DC may improve the predictive efficacy of existing models based on clinical variables for MACCEs in NSTE-ACS patients who underwent complete and successful PCI. These results support the incorporation of relative increase and final coronary physiology combined with DC for risk stratification in NSTE-ACS patients, although validation will require larger prospective studies.

## Data Availability Statement

The datasets used and/or analyzed during this study are available from the corresponding author on reasonable request. Requests to access these datasets should be directed to LY, lileiyu@whu.edu.cn or HJ, hong-jiang@whu.edu.cn.

## Ethics Statement

The studies involving human participants were reviewed and approved by the Ethics Committee of Renmin Hospital of Wuhan University (No. WDRY2021-K078). The Ethics Committee waived the requirement of written informed consent for participation.

## Author Contributions

LY and HJ: substantial contributions to conception and design, data acquisition, and data analysis and interpretation. JW, CL, FG, ZZ, HC, LZ, YW, HZ, ZL, SD, JS, QD, and SX: drafting the article or critically revising it for important intellectual content. JW, CL, and FG: final approval of the version to be published and agreement to be accountable for all aspects of the work in ensuring that questions related to the accuracy or integrity of the work are appropriately investigated and resolved. All authors contributed to the article and approved the submitted version.

## Funding

This work was supported by the National Natural Science Foundation of China (81871486, 81970287, and 82100530).

## Conflict of Interest

The authors declare that the research was conducted in the absence of any commercial or financial relationships that could be construed as a potential conflict of interest.

## Publisher's Note

All claims expressed in this article are solely those of the authors and do not necessarily represent those of their affiliated organizations, or those of the publisher, the editors and the reviewers. Any product that may be evaluated in this article, or claim that may be made by its manufacturer, is not guaranteed or endorsed by the publisher.

## Supplementary Material

Supplementary Figure 1 Comparison of the predictive capacity and accuracy of relative increase and final QFR and DC for MACCEs. Model 5: Model 1 + Post-PCI QFR of the target vessel ≤0.88. Model 6: Model 1 + %QFR increase of the target vessel ≤23%. Model 7: Model 1 + DC 24h ≤2.42 ms.

## Abbreviations

DC: deceleration capacity
DC 24h: 24-hour deceleration capacity
QFR: quantitative flow ratio
ACS: acute coronary syndrome
CAD: coronary artery disease
NSTE-ACS: non-ST-elevation ACS
NSTEMI: acute non-ST-segment elevation myocardial infarction
LAD: left anterior descending artery
LCX: left circumflex artery
RCA: right coronary artery
PCI: percutaneous coronary intervention
MACCEs: major adverse cardiac and cerebrovascular events
ROC: receiver operating characteristic
HR: hazard ratio
CI: confidence interval
ANS: automatic nervous system
SDNN: standard deviation of all normal sinus RR intervals
RMSSD: root mean square successive difference
SDANN: standard deviation average of NN intervals
PNN50: percentage of the number of times that the difference between adjacent normal RR intervals >50 ms over the total number of NN intervals
HF: high-frequency power
LF: low-frequency power.
