# Supplementary figures and images for: Deceleration Capacity Improves Prognostic Accuracy of Relative Increase and Final Coronary Physiology in Patients With Non-ST-Elevation Acute Coronary Syndrome
Source: Front Cardiovasc Med. 2022 Mar 22;9:848499. doi: 10.3389/fcvm.2022.848499 (PMC8980553; doi:10.3389/fcvm.2022.848499)

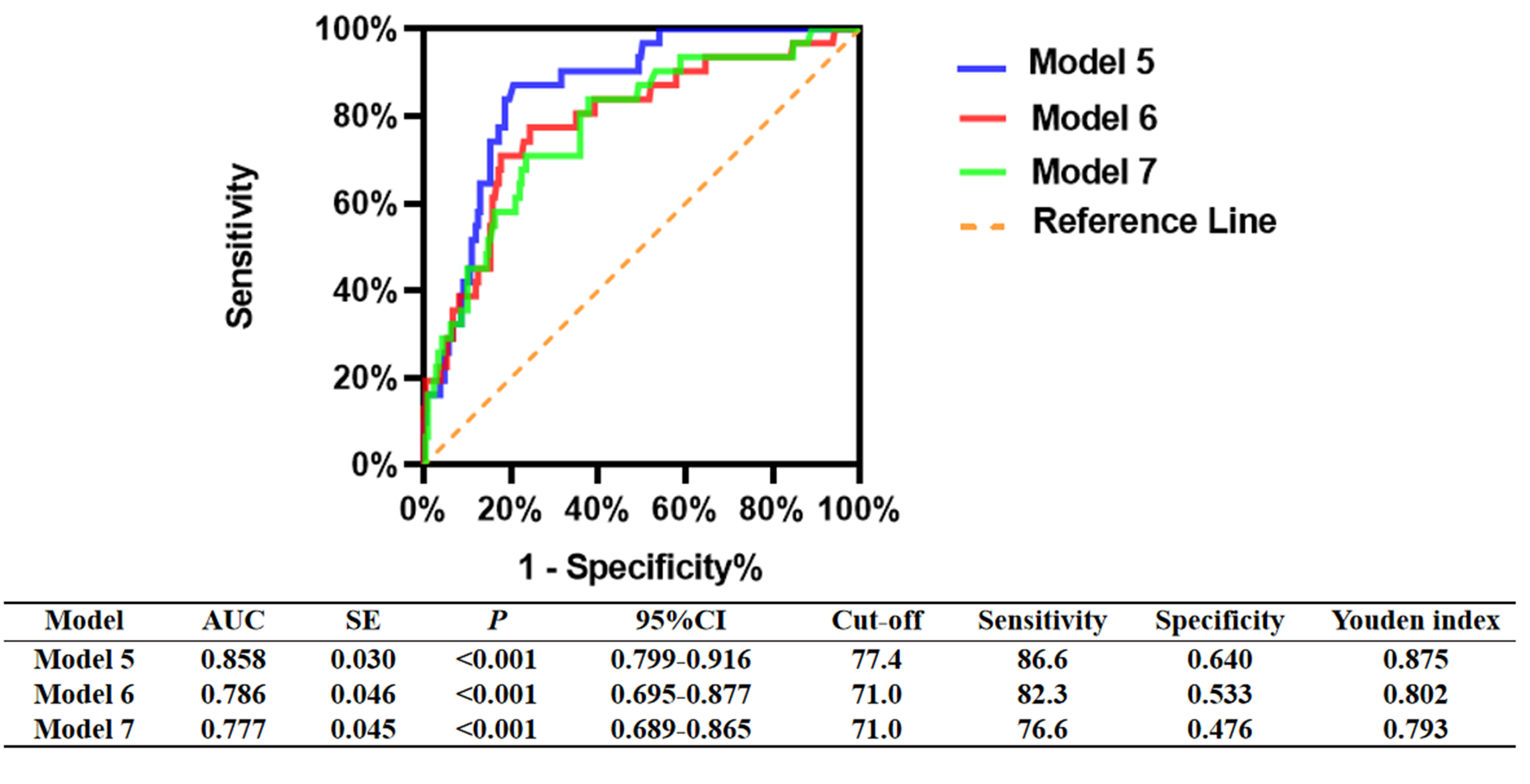

Supplement: Supplementary Figure 1 — Comparison of the predictive capacity and accuracy of relative increase and final QFR and DC for MACCEs. Model 5: Model 1 + Post-PCI QFR of the target vessel ≤0.88. Model 6: Model 1 + %QFR increase of the target vessel ≤23%. Model 7: Model 1 + DC 24h ≤2.42 ms. [file Image_1.TIF]
